# Effect of Feeding Barley, Corn, and a Barley/Corn Blend on Beef Composition and End-Product Palatability

**DOI:** 10.3390/foods10050977

**Published:** 2021-04-29

**Authors:** Wilson Barragán-Hernández, Michael E. R. Dugan, Jennifer L. Aalhus, Gregory Penner, Payam Vahmani, Óscar López-Campos, Manuel Juárez, José Segura, Liliana Mahecha-Ledesma, Nuria Prieto

**Affiliations:** 1 Lacombe Research and Development Centre, Agriculture and Agri-Food Canada, Lacombe, AB T4L 1W1, Canada; wbarraganh@agrosavia.co (W.B.-H.); mike.dugan@canada.ca (M.E.R.D.); jennifer.aalhus@outlook.com (J.L.A.); oscar.lopezcampos@canada.ca (Ó.L.-C.); manuel.juarez@canada.ca (M.J.); jose.seguraplaza@canada.ca (J.S.); 2 Faculty of Agricultural Sciences, University of Antioquia, Medellín 1226, Colombia; Liliana.mahecha@udea.edu.co; 3 Corporación Colombiana de Investigación Agropecuaria (AGROSAVIA), El Nus Research Centre, San Roque 053037, Colombia; 4 College of Agriculture and Bioresources, University of Saskatchewan, Saskatoon, SK S7N 5A8, Canada; greg.penner@usask.ca; 5 Department of Animal Science, University of California, 2201 Meyer Hall, Davis, CA 95616, USA; pvahmani@ucdavis.edu

**Keywords:** barley, corn, blend, eating quality, volatile compounds, fatty acids, beef

## Abstract

This study evaluated the relationship among palatability attributes, volatile compounds, and fatty acid (FA) profiles in meat from barley, corn, and blended (50:50, barley and corn) grain-fed steers. Multiple correspondence analysis with three dimensions (Dim) explained 62.2% of the total variability among samples. The Dim 1 and 2 (53.3%) separated pure from blended grain-fed beef samples. Blended grain beef was linked to a number of volatiles including (E,E)-2,4-decadienal, hexanal, 1-octen-3-ol, and 2,3-octanedione. In addition, blended grain-fed beef was linked to fat-like and rancid flavors, stale-cardboard, metallic, cruciferous, and fat-like aroma descriptors, and negative categories for flavor intensity (FI), off-flavor, and tenderness. A possible combination of linoleic and linolenic acids in the blended diet, lower rumen pH, and incomplete biohydrogenation of blended grain-fed polyunsaturates could have increased (*p* ≤ 0.05) long-chain n-6 fatty acids (LCFA) in blended grain-fed beef, leading to more accumulation of FA oxidation products in the blended than in barley and corn grain-fed meat samples. The Dim 3 (8.9%) allowed corn separation from barley grain beef. Barley grain-fed beef was mainly linked to alkanes and beef positive FI, whereas corn grain-fed beef was associated with pyrazines, in addition to aldehydes related to n-6 LCFA oxidation.

## 1. Introduction

Beef is a valuable food for human nutrition, offering rich contents of available protein, fat, vitamins, and minerals. A European study found that consumption between 75 and 211 g/d of meat contributed to the intake of protein and saturated, monounsaturated, and polyunsaturated fats in a range of 29 to 41%, 19 to 24%, 23 to 28%, and 11 to 20%, respectively, as well as contributing with a variety of vitamins and minerals such as B12 vitamin (29–37%) and zinc (27–37%) [1]. However, beyond health interest, beef consumption is strongly influenced by overall consumer liking, with flavor explaining between 38% and 48% of the variability [2,3,4].

Beef flavor and other sensory attributes are influenced by ante and post-mortem factors related to genetics, feeding systems, ageing/storage conditions, and cooking methods [5,6,7]. Volatile compounds responsible for beef flavor originate from water-soluble substances and lipid precursors resultant from the Maillard reaction and thermal lipid oxidation [8]. For beef, volatiles include alkanes, aldehydes, ketones, alcohols, furans, esters, and pyrazines as commonly reported, among other volatiles [9]. These compounds alone or together can positively or negatively stimulate the complex system of consumer senses located in the tongue, mouth, and nasal cavity to develop an opinion regarding its acceptability [8].

The study of beef volatiles is a tool that can be used to interpret complex flavors and anticipate consumer satisfaction, as volatile organic compounds have been associated with consumer perception and satisfaction [10]. For instance, reviews by Mottram [11] and Calkins and Hodgen [12] indicate pyrazines, furans, and alkanes can be related to pleasant and roasted/grilled flavor in beef, whereas some aldehydes and alcohols can contribute to some off-flavors. In this context, knowing the effect of dietary manipulation on volatile profiles could help anticipate consumer beef acceptance [3].

On the Canadian prairies, finishing cattle on barley grain-based diets is the norm [13]. However, corn grain finishing is growing due to development of new varieties adapted to low-luminosity and low-temperature conditions [14]. Additionally, barley and corn blended diets have been proposed to support least-cost ration formulation, and take advantage of greater rates of starch bypass from the rumen, which could improve energetic efficiency and marbling fat deposition [15,16].

Canadian and Japanese consumers have shown a preference for barley over corn grain-fed beef [17]. O’Quinn et al. [3] reported differences in volatile profiles from barley or corn grain-fed beef. However, Jeremiah et al. [18] and McEwen et al. [19] reported no differences in flavor between corn and barley grain-fed beef. Hence, there is some controversy over effects of grain type fed on flavor and their association with volatile profiles, and no data are available for comparing barley, corn, and blended grain-fed beef. Additionally, discrepancies in the fatty acid composition from barley and corn grain-fed beef have also been reported among studies [3,17,20], fatty acids being key precursors of volatile compounds [21].

The objective of the present study was to evaluate the effect of feeding barley, corn, and a barley/corn blend on descriptive sensory attributes, volatile compounds, and flavor and fatty acid profiles from beef, following a qualitative approach.

## 2. Materials and Methods

### 2.1. Animals, Diets, and Collection of Samples

A complete description of the animals, diets, and butchering process used herein is available in Johnson et al. [13]. The research protocol for this study was preapproved by the University of Saskatchewan Animal Research Ethics Board (protocol 20100021), according to the guidelines of the Canadian Council on Animal Care (Ottawa, ON, Canada). In short, 288 commercial crossbred steers (464 ± 1.7 kg) were randomly assigned to 24 pens (12 steers/pen) at the University of Saskatchewan. Four pens were randomly assigned to six treatments following a factorial (2 × 3) design of silage source (corn or barley, 8% dry matter—DM) and grain source: barley (86% DM), corn (85% DM), or blend (50:50 barley and corn, 85% DM). Silage was balanced across grain treatments, and all diets included the same minerals and vitamins and were isoproteic. Following 89 days on feed, steers (623 ± 86 kg) were taken to a federally inspected abattoir (Cargill Meat Solution, High River, AB, Canada) and slaughtered according to Canadian Council on Animal Care principles and guidelines [22].

For this study, a total of 85 steers were randomly selected (4 steers/pen) considering only the grain-fed source (27 for corn, 29 for barley, and 29 for blend treatment). After slaughter, the *longissimus thoracis* (LT) between the 6th and 12th ribs (bone-in ribeye) from each carcass was collected, transported in a refrigerated vehicle (2–4 °C) to the Lacombe Research and Development Centre (Lacombe, AB, Canada), and aged in a cooler at 2 °C, 0.5 m·s^−1^ of wind speed, and 80% of relative humidity for 15 days. Following ageing, four 25 mm steaks (LT between the 8th and 12th ribs) were taken from each bone-in ribeye, trimmed of all subcutaneous and seam fat, and assigned respectively to fatty acids, volatile compounds, flavor profile, and descriptive sensory analyses.

### 2.2. Sensory Analyses

Steaks designated for descriptive sensory and flavor profile analyses were cooked after being thawed for 24 h at 4 °C. A 10 cm spear point Type T thermocouple probe (Wika Instruments, Edmonton, AB, Canada) was inserted into the center of the steak and connected to a Hewlett Packard HP34970A Data Logger (Hewlett Packard Co., Boise, ID, USA) to monitor the internal temperature of the steaks while cooking. On a Garland grill (Model ED30B, Condon Barr Food Equipment Ltd., Edmonton, AB, Canada) that was preheated to 210 °C, steaks were grilled to an internal temperature of 35.5 °C, flipped, and were taken off when they reached 71 °C. After removal from the grill, steaks were cooled for 3 min, and then each steak was subsampled by cutting 1.3 × 1.3 × 1.3 cm cubes, avoiding areas with high levels of connective tissue or fat. Prior to sensory analysis, the temperature of the steak cubes was equilibrated by putting samples in covered glass containers in a circulating water bath (68 °C). Samples were presented to a trained expert, nine-member meat evaluation panel in a balanced design with sample assignment determined using Compusense 5 Software, version 4.6 (Compusense Inc., Guelph, ON, Canada).

The panelists rated the following attributes from steak samples for descriptive attribute sensory analyses: initial and overall tenderness, initial and sustained juiciness, beef flavor and off-flavor intensity, amount of connective tissue, and residual mouth coating. nine-point descriptive scales were used to assign the scores: 9 = extremely tender, extremely juicy, extremely intense beef flavor, extremely bland off-flavor, no connective tissue, and no residual mouth coating; 1 = extremely tough, extremely dry, extremely bland beef flavor, extremely intense off-flavor, extremely abundant connective tissue, and extremely abundant residual mouth coating.

The AMSA flavor lexicon was used for flavor profile analysis [20]. Samples were evaluated using a 15 cm line scale with standard reference points for detected tastes (sweet, sour, bitter, salty, and umami), aromas, and flavors (brown-roasted, beef identity, cruciferous, oily, grainy, bloody-serumy, corn, liver-like, sour-dairy, green-hay, burnt, barnyard, buttery, metallic, stale-cardboard, other, and unidentified) (0 = none; 15 = extremely intense).

Paid panelists, who had served as trained experts for an average of 6 years, were recruited, screened, and trained [23] to exclusively evaluate meat samples. The guidelines set forth by the AMSA were used to evaluate and monitor panelists’ performance [20]. For each session, the number of steaks evaluated included 6 for descriptive sensory analyses and 3 for flavor profile analyses. Four sessions were conducted per day in total: 2 in the morning and 2 in the afternoon, with a 20 min break between sessions in the morning and afternoon. All panel evaluations were performed in partitioned booths that were well-ventilated and illuminated by 180 lux green lighting. Unsalted soda crackers and distilled water were supplied to cleanse the palate of residual flavor notes between samples [24].

### 2.3. Volatile Compounds

Analysis of beef volatile compounds was performed as outlined in Ruan et al. [9]. Briefly, steaks were grilled to a final temperature of 71 °C as described above. Steaks were then ground for 15 s at 10 × 1000 rpm with Grindomix GM200 (Rest GmbH, Haan, Germany) and subsampled in triplicate (1 g each) for stir bar sorptive extraction. This was coupled with thermal desorption–gas chromatography–mass spectrometry analyses (SBSE-TD-GC-MS). Each subsample was placed into a 10 mL sample vial with 8 mL of extraction solution (75% saturated NaCl with 25% MeOH, *v*/*v*). A commercial sorptive stir bar (TwisterTM GERSTEL GmbH & Co.KG, Mülheiman der Ruhr, Germany) was added to each vial to agitate the meat slurry for 120 min at 35 °C × 1000 rpm on a Gerstel Twister^®^ stir plate (Gerstel GmbH & Co. KG, Mülheiman der Ruhr, Germany). Stir bars were then thermally desorbed by programming the TDS 2 from 40 °C (held for 1 min) to 200 °C (held for 5 min) at 60 °C/min. The desorbed compounds were cryofocused in the CIS 4 at −120 °C. Following desorption, the CIS 4 was programmed from 40 to 275 °C (held for 5 min) at 12 °C/s to inject the trapped compounds onto the analytical column. The separations were executed on an HP-5 MS fused-silica capillary column (30 m × 0.25 mm I.D., 0.25 μm film thickness, Agilent Technologies, Santa Clara, CA, United States). Oven temperature was programmed from 50 °C (held for 1 min) to 100 °C (held for 2 min) at 10 °C/min, then to 280 °C (held for 1 min) at 30 °C/min. The carrier gas was helium with a flow rate of 1.2 mL/min. Volatile compounds were tentatively identified using a mass spectral library in NIST 08 (NIST 08 version 2.0) requiring a match factor over 85 with the retention index for all the volatile compounds. The base peak (*m*/*z*) of each volatile was then standardized to the internal reference peak (Nonanal).

### 2.4. Fatty Acid Analysis

Intramuscular lipid extraction and fatty acid analysis were conducted as outlined in Vahmani et al. [25]. In short, chloroform−methanol (2:1, *v*/*v*) was used to extract subsamples from steaks between the 11th and 12th ribs. Acid (5% methanolic HCl) and base (0.5 N sodium methoxide) were then used to methylate an aliquot of lipids from each tissue. Fatty acid methyl esters (FAME) were then analyzed using a CP-3800 gas chromatograph equipped with a 100 m CP-Sil 88 fused capillary column (Varian Inc., Mississauga, ON, Canada) with *c*10-17:1 methyl ester (Nu-Check Prep Inc., Elysian, MN, USA) as the internal standard. To quantify the FAME, chromatographic peak area and internal standard-based calculations were employed. 

### 2.5. Statistical Analyses

Principal component analysis (PCA) was applied to all the volatile compounds tentatively identified in the samples from this study to select the most important compounds from the dataset. The volatile compounds were selected based on their loadings in the PCA and then binarized with a threshold of 0.4 times internal reference peak: compounds > 0.4 times internal reference peak were considered as present (Volatile_y), and anything else as absent (Volatile_n). The descriptive sensory attributes and flavor profile datasets were categorized as well. Descriptive sensory attributes with an average rating ≥6 were categorized as positive (Sensory attribute_Positive), 5 = neither positive or negative (Sensory attribute_Neither), and <5 as negative (Sensory attribute_Negative). A flavor descriptor was present when the average of panelists’ rates was higher than 1 (aroma/taste/flavor descriptor—A/T/F y), and anything else was considered absence (aroma/taste/flavor descriptor—A/T/F n). 

The descriptive sensory attributes, flavor profile, and volatile compounds from barley, corn, and blended grain-fed beef were submitted to multiple correspondence analysis (MCA), using a FactoMiner package [26] in the software R-Project (version 3.6.1., 2019, Team Core R, Vienna, Austria). Additionally, to improve insight about fatty acid and volatile compound relationships, a canonical correlation analysis (CCA) was performed using a CCA package in the software R-Project (version 3.6.1., 2019). The results were presented using biplot, structure correlation, and weights in representative dimensions of CCA [27]. Moreover, fatty acid analyses and sensory attributes were conducted using the MIXED procedure of SAS (Version 9.2 Institute Inc., Cary, NC, USA). In fatty acid analysis, grain was used as a fixed effect, and pen as the random effect. For sensory analysis, grain was used as a fixed effect, and pen, trained panelist and session for sensory analysis, as the random effect. In rejection of the null hypothesis, the least-square means difference was conducted by the PDIFF statement with alpha = 0.05. Preliminary analyses showed no effect of silage or silage grain interaction either on sensory attributes or on fatty acid and volatile profiles, and, hence, only the effect of grain type was evaluated in this study.

## 3. Results and Discussion

In this study, a total of 162 volatile compounds were tentatively identified in meat samples from barley, corn, and blended grain-fed steers. After applying a PCA with all the volatiles, five principal components (PC variance > 1) were retained, explaining 84.5% of the total variance. In these PCs, the volatile compounds with a loading higher than 0.2 in absolute value were selected, resulting in a total of 22 tentatively identified volatile compounds considered for this study (Table 1). The rest of the volatile compounds not selected are presented in the Supplementary Table S1. Among volatiles selected, the most significant groups were alkanes (31.8%), aldehydes (18.1%), ketones (13.6%), alcohols (9%) derived from lipids, and pyrazines (9%) originated from water-soluble compounds resulting from lipid thermal and Maillard reactions [28].

**Figure 1:**
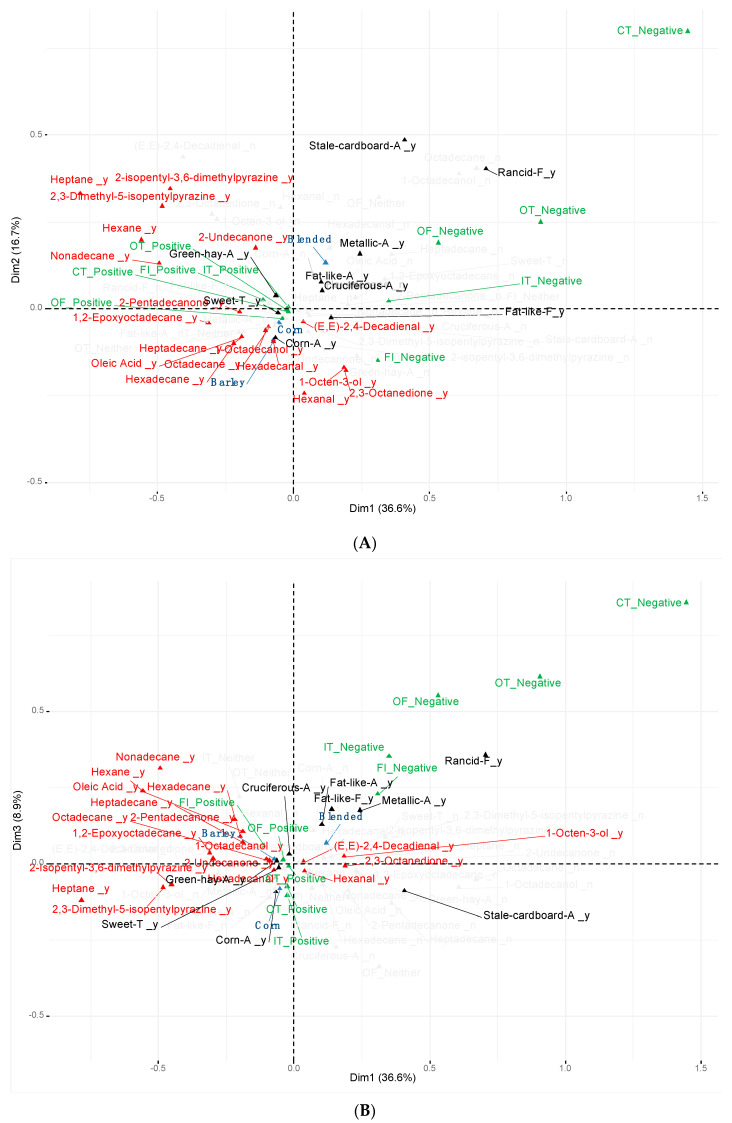
Multiple correspondence analysis among descriptive sensory attributes, flavor profile, and volatile compounds from barley, corn, and blended grain-fed beef. Blue: grain diet; red: volatile compounds; black: aroma/taste/flavor descriptors; green: descriptive sensory attributes. (**A**) Dimensions 1 and 2. (**B**) Dimensions 1 and 3.

A full description of the descriptive sensory and flavor profile (aromas, tastes, and flavors) attributes from barley, corn, and blended grain-fed beef are presented in the Supplementary Table S2. The MCA performed among sensory attributes, flavor profile, and volatile compounds from barley, corn, and blended grain-fed beef achieved 62.2% of the total variability in three dimensions (Figure 1). This total variability was reached by considering the variables showing a high contribution to the construction of the dimensions. Hence, only those variables explaining a high variability are presented in Figure 1.

The first and second dimensions (Dim 1 and Dim 2) of the MCA explained 36.6% and 16.7% of the variability, respectively (Figure 1A). Both Dim 1 and 2 contributed to discriminate pure grains (barley or corn) against the blended grain-fed beef based on descriptive sensory attributes, flavor profile, and volatile compounds. The barley and corn grain-fed categories were located in the lower-left quadrant, whereas the blended grain-fed beef was observed in the upper-right quadrant. Specifically, the positive axis of Dim 1 was associated with the blended grain-fed category and lipid products such as aldehydes [(E, E)-2,4-decadienal and hexanal] and alcohols (1-octen-3-ol). These volatile compounds contribute to beef flavor characteristic [29]. However, in their highest concentrations, these volatiles are associated with adverse lipid oxidation odors such as rancidity, fishy, and grassy [8,30,31]. Additionally, the blended grain-fed category was also associated by Dim 1 with 2,3-octanedione that has an oxidized fat and warmer over flavor derived from lipid oxidation [32]. Indeed, the positive space of the Dim 1 was also characterized by the presence of stale-cardboard, fat-like, metallic, and cruciferous aromas, fat-like and rancidity flavor descriptors, and the negative categories for off-flavor (from slightly to extremely intense off-flavor) and beef flavor intensity (from slightly to extremely bland beef flavor). The association among aroma and flavor descriptors related to oxidation, low beef flavor intensity, and the above-mentioned volatile compounds was previously described by Larick and Turner [30] and Kerth and Miller [8] in beef. Burnett et al. [21] and Therkildsen et al. [33] described a negative association between polyunsaturated fatty acids (PUFA) and beef flavor intensity and overall palatability due to fatty acid oxidation. In this study, the CCA between fatty acids and volatile compounds showed an association among the C18:2 n-6 ratio (r = −0.432), n-6 FA group (r = −0.397), PUFAs (r = −0.385), C22:6 n-3 (r = −0.380), PUFA/saturated fatty acids (SFA) ratio (r = −0.334), hexanal (r = −0.422) 2,3-octanedione (r = 0.3462), and 1-octen-3-ol (r = 0.2866) in the negative space of the Dim 1 (Roy’s statistics test *p* < 0.001). These results agree with Calkins and Sullivan [34] and Larick and Turner [30], who reported hexanal and 1-octen-ol are related to lipid oxidation from PUFAs.

The meat from blended grain-fed cattle was also characterized by negative categories for initial tenderness, overall tenderness, and connective tissue (tougher meat and with an abundant connective tissue), with overall tenderness and connective tissue being located in the positive space of both Dim 1 and 2, and initial tenderness in the positive and negative space of Dim 1 and 2, respectively. This agrees with Legako et al. [35], who reported a negative correlation between aldehydes and consumer palatability scores for overall liking, tenderness, and overall tenderness, and also desirable flavor descriptors such as beef identity, bloody-serumy, brown-roasted, and umami. However, in contrast to our results, these authors also reported a negative correlation between aldehydes and fat-like flavor descriptor. Starowicz and Zieliński [36] suggested that the Maillard reaction could be related to tenderness improvement via alteration of protein cross-linking based on the amino acids involved. This hypothesis was previously corroborated by Sun et al. [37], who used Maillard reaction modificated proteins from mechanically deboned chicken prepared at 90 °C to fabricate Cantonese sausages with less hardness and chewiness texture. The present study may corroborate this theory due to the association between the negative categories for initial and overall tenderness and connective tissue and the absence of volatiles from the Maillard reaction in the positive space of the Dim 1.

**Figure 2:**
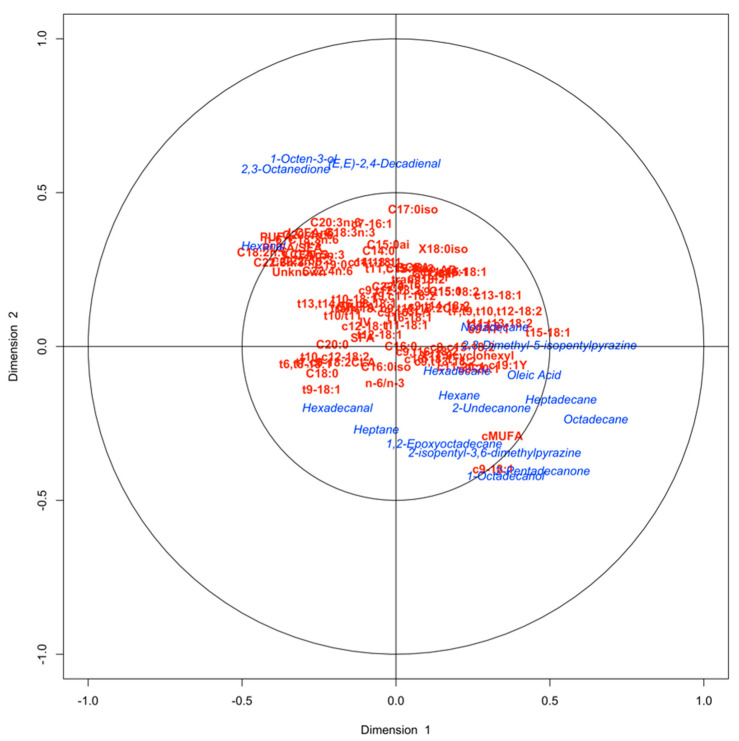
Canonical correlation between fatty acids (red) and volatile compounds (blue) from barley, corn, and blended grain-fed beef.

Unlike blended grain, the negative space of Dim 1 and Dim 2 (lower-left quadrant) was associated with both barley and corn grain-fed beef and associated with the presence of (1) alkanes (hexadecane, heptadecane, and octadecane) and ketones (2-pentadecanone), which have been related to pleasant flavors such as meaty and brown-roasted [38]; (2) oleic acid positively related to desirable beef palatability; (3) the positive category for off-flavor intensity (from slightly bland to no off-flavor), sweet taste, and corn aroma. Barley and corn-fed beef were also linked to hexadecanal, which has been related to fatty odor, however, this aldehyde has a small contribution to meat flavor [31]. Additionally, some pyrazine compounds (2,3-dimethyl-5-isopentylpyrazine and 2-isopentyl-3,6-dimethylpyrazine) and alkanes (nonadecane, hexane, and heptane) linked to meaty and roasted flavor [11,12,28], ketones (2-undecanone), a positive category for beef flavor intensity (from slightly to extremely intense beef flavor), and green-hay aroma were associated to pure grain-fed meat by the negative axis of the Dim 1. On the other hand, the positive space of Dim 1 of the CCA (Figure 2) showed an association between fatty acids such as C18:1t15 (r = 0.494), C19:1 (r = 0.362), cMUFAs (r = 0.344), and C18:1c13 (r = 0.343), and volatile compounds mentioned above, such as octadecane (r = 0.626), heptadecane (r = 0.512), 2,3-dimethyl-5-isopentylpyrazine (r = 0.496), and 2-pentadecanone (r = 0.451). These results agree with those of Mottram [11], who reported that alkanes come from the oxidation of long-chain fatty acids and, together with pyrazines and other volatile compounds, contribute to the pleasant beef flavor [8]. Likewise, O’Quinn et al. [3] and Hwang and Joo [39] reported an association between MUFAs and beef flavor desirability and overall palatability in beef. Overall, the volatile profiles associated with barley and corn grain-fed beef in this study are in agreement with that found by O’Quinn et al. [3]. Moreover, both barley and corn grain-fed meat were associated with positive initial and overall tenderness and connective tissue categories, although to a lesser extent due to their proximity to the origin of both Dim 1 and 2. 

The reason why meat from blended grain-fed cattle was associated with aroma and flavor descriptors related to lipid oxidation, negative categories for descriptive sensory attributes, and volatile compounds related to PUFA oxidation could be related to differences in the total fatty acids (mg/g of LT) and the fatty acid composition (% of total fatty acids) (Table 2). Many of the LT fatty acids for the blended grain-fed beef are between or close to the values for either barley- or corn-fed beef (n-3, atypical dienes, cisMUFA, transMUFA, and SFA), and would thus not explain why feeding blended grain would enhance oxidation. There was some indication that feeding the blend might enhance ∑ PUFAs, ∑ n-6 fatty acids, and specifically, 18:2n-6 in LT fatty acids, as their percentages were numerically greater than in barley- or corn-fed meat, but differences did not reach significance. Significant differences were, however, found for 18:2n-6 elongation and desaturation products (i.e., 20:3n-6, *p* < 0.05; 20:4n-6, *p* = 0.05), which would support greater oxidation potential for blended grain-fed LT as susceptibility to oxidation increases geometrically as the number of double bonds increases. In addition, long-chain PUFAs are preferentially incorporated into cell membrane phospholipids, which is where initiation of fatty acid oxidation is thought to occur [40]. The reason for elevated n-6 fatty acids when feeding the blend are not related to the dietary supply, but instead likely relate to effects on rumen pH. Lower rumen pH was found in blended compared with barley and corn grain-fed steers in the present study, as previously reported by Johnson et al. [41]. Under these conditions, lipolysis of dietary lipids could have been inhibited [42], reducing PUFA biohydrogenation by rumen bacteria, and allowing for greater bypass of n-6 fatty acids. 

The differences in descriptive sensory attributes and flavor profiles between meat samples from blended and pure (barley or corn) grain-fed cattle found in this study are in contrast to those reported by Miller et al. [43], who found no differences in the eating quality between meat samples from those feeding regimes. These discrepancies between studies could be due to the lack of effect of grain type or blend on beef fatty acid composition, which may relate to lower levels of grain in their diets and differences in grain processing, and limited statistical power due to low number of experimental units (n = 6). In the present experiment, both corn and barley were dry-rolled, whereas Miller et a. [43] steam-rolled corn and crimped barley, which may have impacted rumen fermentation rates and pH.

When the whole dataset of sensory attributes, flavor profile, and volatile compounds were represented on an XY plane according to the Dim 1 and 3 (Figure 1B), Dim 3 explained 8.9% of variability among these variables for meat samples from barley, corn, and blended grain-fed cattle. Similar to that observed in the bi-dimensional plane described by Dim 1 and 2, the blended grain-fed beef was located in the upper-right quadrant and was associated with undesirable aromas, flavors, and sensory attributes, and volatiles originating from lipid oxidation. However, the Dim 3 allowed the quadrant separation of meat samples from each pure grain-finished treatment. The upper-left quadrant of MCA linked barley grain-fed beef to a combination of alkanes (nonadecane, hexadecane, hexane, heptadecane, and octadecane), epoxide (1,2-epoxyoctadecane), ketones (2-pentadecanone and 2-undecanone), and oleic acid, which were previously described by Calkins and Hodgen [12] and Mottram [11] as a combination of volatiles that produce a pleasant meat and roasted flavor. Indeed, positive categories for beef flavor intensity (from slightly intense to extremely intense) and off-flavor (from slightly bland to none) as well as green-hay and cruciferous aromas presented in the same quadrant.

The negative space for Dim 3 related the meat samples from corn-fed cattle to volatile compounds such as pyrazines (2,3-dimethyl-5-isopentylpyrazine and 2-isopentyl-3,6-dimethylpyrazine), alkane (heptane), and ketone (2,3-octanedione), as well as to positive sweet taste and corn aroma. However, corn grain-fed beef was also associated with hexadecanal and hexanal. As previously mentioned, these aldehydes come from the oxidation of n-6 fatty acids [30,35], and, in this study, corn had higher (*p* < 0.05, Table 2) n-6/n-3 ratio than barley grain-fed beef samples. Similar results were reported by Vahmani et al. [20] in subcutaneous fat samples from the same animals. In contrast, Brassard et al. [44] reported lower n-6/n-3 ratio in meat from barley and corn concentrate-fed goats, probably due to hay ad libitum access and different concentration of linolenic intake. As previously mentioned, hexadecanal has been described as a fatty odor contributor with low participation in flavor development [31]. Hexanal is a common volatile found when feeding grain-based diets [45], related to grassy, leafy, and green flavor descriptors, and with low odor threshold [8,28,38]. However, in higher concentrations, hexanal has been previously described as an indicator of meat flavor deterioration [46]. The association of meat from corn-fed steers with hexanal found in this study is in agreement the with the findings of O’Quinn et al. [3], who found higher concentrations of hexanal in meat from corn-fed compared with barley-fed steers. In contrast to the positive association of barley grain-fed beef with beef flavor intensity, no flavor characteristics were related to corn grain-fed beef in this study. This, in part, disagrees with the findings of McEwen et al. [19], who did not report differences in beef flavor intensity between meat from corn-fed steers and barley grain-fed steers, probably due to the different cattle breed (Angus) and lower barley participation in diet composition (70% DM). Nevertheless, Jeremiah et al. [18] found no differences in beef flavor intensity from crossbred cattle fed barley and corn in a concentration similar to that used in this study. Differences in the taste panel (semitrained panel vs. trained panel) and more sophisticated statistical analyses used in the present study could have also contributed to the differences between studies.

Corn grain-fed beef was also associated (negative space of Dim 1 and 3) with positive categories for initial and overall tenderness (from slightly to extremely tender) and connective tissue (from slight to none). These results agree with the findings of Wismer et al. [17], who reported higher initial and overall tenderness in beef from corn- compared with barley-fed cattle. In contrast, several studies have found no differences in descriptive sensory attributes, such as tenderness and amount of connective tissue, evaluated by a trained panel in beef from barley- and corn-fed cattle [18,19,47] despite using cattle breed, grain concentration, and storage time and thawing/cooking conditions of meat samples similar to those used in the current study. Nevertheless, recent advancements in statistical methods could have, in part, contributed to the discrepancies among studies.

## 4. Conclusions

Use of integral analysis of MCA was able to separate barley and corn from blended grain-fed beef based on aroma/flavor profile, descriptive sensory attributes, and volatile compounds. The fatty acid profile of the meat samples suggested an influence of barley and corn in blended grain-fed beef on n-6 LCFA deposition, which could have increased their oxidation potential. This effect was supported by some aldehydes and alcohols from PUFA oxidation, undesirable aromas and flavor descriptors, and negative categories for descriptive sensory traits associated with the blended grain-fed beef samples. Barley and corn grain-fed beef were also differentiated, although with lower explained variance in the MCA; barley grain-fed beef was associated with volatile compounds originating a pleasant beef flavor, whereas corn grain-fed beef was linked to some volatile compounds from lipid oxidation. However, corn grain-fed beef was more associated with positive categories for tenderness. Nevertheless, apart from the positive association of beef flavor intensity with barley grain-fed beef, no flavor descriptors were associated with either barley or corn grain-fed beef, which may suggest that the different volatiles associated with barley and corn grain-fed beef in this study did not translate into differences in meat flavor detected by the trained panelists. Hence, a further quantitative approach to understand the volatile thresholds and their influence on meat palatability would be warranted in order to maximize the potential of volatile compounds to anticipate consumer satisfaction. In addition, even though feeding blended grain-fed diets may at times be economically feasible, the interaction between different fed grains leading to oxidative instability may be an unanticipated outcome, and thus deserves further attention.

## Figures and Tables

**Table 1:** Mean of standardized base peak of selected volatile compounds from barley, corn, and blended grain-fed beef samples.

Volatile Compounds	Barley	Blended	Corn
(E,E)-2,4-Decadienal	1.837978	1.80720552	1.55708039
(Z)-7-Hexadecenal	5.30218346	4.48371819	5.38537755
1-Octadecanol	0.96836213	0.86885953	0.99385898
1-Octen-3-Ol	0.5153148	0.44414982	0.42838899
1,2-Epoxyoctadecane	0.54637487	0.33669352	0.55077623
2-Isopentyl-3,6-Dimethylpyrazine	0.29760656	0.40774939	0.36947751
2-Pentadecanone	0.49167099	0.44082738	0.49833478
2-Undecanone	0.24967149	1.22870434	0.40275022
2,3-Dimethyl-5-Isopentylpyrazine	0.3877261	0.19724228	0.28495414
2,3-Octanedione	0.59298402	0.51187674	0.52339562
Heptadecane	0.74794743	0.56457323	0.55966006
Heptane	0.14459152	0.12024081	0.06092762
Hexadecanal	0.85396753	0.53034371	0.87460321
Hexadecane	0.38918876	0.37397273	0.35616684
Hexanal	0.55325571	0.39576164	0.53619463
Hexane	0.34493859	0.19225675	0.17337753
Methyl Oleate	2.29272729	1.82602211	1.73166007
n-Hexadecanoic Acid	0.07806632	0.07472788	0.02894254
Nonadecane	0.13397839	0.229666	0.06135028
Octadecane	1.08206796	0.92397752	0.89970811
Oleic Acid	0.57770987	0.56190167	0.39991017
Undecane	0.05524978	0.23207027	0.39906141

**Table 2:** Fatty acid composition ^a^ in longissimus thoracis samples from barley, corn, and blended grain-fed cattle.

	Barley	Blend	Corn	*p*-Value ^1^
∑ Total Fatty Acids (mg/g of tissue)	40.06 ± 2.08	40.91 ± 2.01	46.34 ± 2.13	0.08
Fatty Acids (% of total fatty acids)				
∑ PUFAs ^b^	4.83 ± 2.03	5.28 ± 0.22	4.72 ± 0.24	0.18
∑ n-3	0.69 ± 0.04 ^A^	0.65 ± 0.04 ^A^	0.52 ± 0.04 ^B^	0.01
C18:3n-3	0.26 ± 0.01 ^A^	0.24 ± 0.01 ^A^	0.20 ± 0.01 ^B^	0.01
C20:5n-3	0.11 ± 0.01 ^A^	0.11 ± 0.01 ^A^	0.07 ± 0.01 ^B^	0.02
C22:5n-3	0.28 ± 0.02 ^A^	0.28 ± 0.02 ^A^	0.22 ± 0.02 ^B^	0.02
C22:6n-3	0.04 ± 0.00	0.03 ± 0.00	0.03 ± 0.00	0.16
∑ n-6 ^c^	4.15 ± 0.20	4.63 ± 0.19	4.20 ± 0.20	0.16
C18:2n-6	2.81 ± 0.13	3.21 ± 0.13	3.03 ± 0.14	0.10
C20:3n-6	0.25 ± 0.01 ^A,B^	0.26 ± 0.01 ^A^	0.21 ± 0.01 ^B^	0.04
C20:4n-6	0.91 ± 0.06 ^A,B^	0.96 ± 0.05 ^A^	0.77 ± 0.06 ^B^	0.05
∑ Atypical Dienes ^d^	0.45 ± 0.01 ^A^	0.40 ± 0.01 ^B^	0.38 ± 0.01 ^B^	<0.0001
*t*11,*c*15-18:2	0.10 ± 0.01 ^A^	0.06 ± 0.01 ^B^	0.05 ± 0.01 ^B^	0.00
∑ Conjugated Linoleic Acids ^e^	0.29 ± 0.01	0.30 ± 0.01	0.30 ± 0.01	0.55
*c*9,*t*11-18:2	0.18 ± 0.01	0.18 ± 0.01	0.19 ± 0.01	0.54
∑ *cis* MUFAs ^f^	44.50 ± 0.50	44.90 ± 0.48	45.10 ± 0.50	0.63
*c*9-16:1	3.31 ± 0.10	3.26 ± 0.10	3.04 ± 0.10	0.09
*c*9-18:1	36.33 ± 0.43	37.21 ± 0.42	37.80 ± 0.44	0.07
∑ *trans* MUFAs ^g^	2.79 ± 0.13	2.68 ± 0.13	2.67 ± 0.13	0.77
*t*10-18:1	1.31 ± 0.10	1.13 ± 0.09	1.07 ± 0.10	0.20
*t*11-18:1	0.51 ± 0.02	0.53 ± 0.02	0.57 ± 0.02	0.15
∑ SFA ^h^	45.62 ± 0.47	45.10 ± 0.45	45.64 ± 0.48	0.62
C16:0	26.68 ± 0.30	26.36 ± 0.28	26.18 ± 0.30	0.50
C18:0	13.9 ± 0.26 ^B^	14.0 ± 0.25 ^B^	14.8 ± 0.27 ^A^	0.02
n-6/n-3	6.22 ± 0.21 ^C^	7.26 ± 0.19 ^B^	8.32 ± 0.21 ^A^	<0.0001

^1^ Different uppercase letter ^A,B,C^ in the same row means significant difference according to Tukey’s test (*p* < 0.05). ^a^ Least-square means ± standard error of mean. ^b^ Σ PUFAs = sum of polyunsaturated fatty acids (∑ n-6 + ∑ n-3). ^c^ Σ n-6 = C18:2n-6; C18:3n-6; C20:2n-6; C20:3n-6; C20:4n-6; C22:4n-6. ^d^ Σ Atypical dienes = *c*9,*t*14-18:2; *c*9,*t*13-18:2; 17:0-cyclohexyl; *c*9,*t*15-18:2; *c*9,*t*12-18:2; *t*11,*c*15-18:2; *c*9,*t*16-18:2; *c*9,*c*15-18:2. ^e^ Σ Conjugate linoleic acid = *t*7,*c*9-18:2 (CLA); *c*9,*t*11-18:2 (CLA); *t*9,*c*11-18:2; *t*10,*c*12-18:2; *t*11,*t*13-18:2; *t*7,*t*9-t10,*t*12-18:2. ^f^ Σ *c*MUFAs = sum of monounsaturated cis fatty acids (*c*9-14:1; *c*7-16:1; *c*9-16:1; *c*11-16:1; *c*13-16:1; *c*9-17:1; *c*9-18:1; *c*11-18:1; *c*12-18:1; *c*13-18:1; *c*14-18:1; *c*15-18:1; *c*16-18:1; *c*-19:1-Y; *c*9-20:1; *c*11-20:1). ^g^ Σ *trans* MUFAs = sum of monounsaturated trans fatty acids (*t*6-*t*8-18:1; *t*9-18:1; *t*10-18:1; *t*11-18:1; *t*12-18:1; *t*13-*t*14/*c*6-*c*8-18:1; *t*15-18:1; *t*16-18:1). ^h^ Σ Sum SFA = sum of saturated fatty acid (C14:0; C15:0; C16:0; C17:0; C18:0; C19:0; C20:0; C22:0).

## Supplementary Materials

The following are available online at https://www.mdpi.com/article/10.3390/foods10050977/s1, Table S1: Mean of standardized base peak of non-selected volatile compounds from barley, corn and blended grain-fed beef samples. Table S2: Mean and standard deviation of sensory descriptive and flavour profile attributes from barley, corn and blended grain-fed beef samples.

## References

[1] Cocking C., Walton J., Kehoe L., Cashman K.D., Flynn A. (2020). The role of meat in the European diet: Current state of knowledge on dietary recommendations, intakes and contribution to energy and nutrient intakes and status. Nutr. Res. Rev..

[2] Drey L., Legako J., Brooks J., Miller M., O’quinn T. (2017). The contribution of tenderness, juiciness, and flavor to overall consumer beef eating experience. Meat Muscle Biol..

[3] O’Quinn T.G., Woerner D.R., Engle T.E., Chapman P.L., Legako J.F., Brooks J.C., Belk K.E., Tatum J.D. (2016). Identifying consumer preferences for specific beef flavor characteristics in relation to cattle production and postmortem processing parameters. Meat Sci..

[4] Liu J., Ellies-Oury M.P., Chriki S., Legrand I., Pogorzelski G., Wierzbicki J., Farmer L., Troy D., Polkinghorne R., Hocquette J.F. (2020). Contributions of tenderness, juiciness and flavor liking to overall liking of beef in Europe. Meat Sci..

[5] Khan M.I., Jo C., Tariq M.R. (2015). Meat flavor precursors and factors influencing flavor precursors-A systematic review. Meat Sci..

[6] Marrone R., Salzano A., Di Francia A., Vollano L., Di Matteo R., Balestrieri A., Anastasio A., Barone C.M.A. (2020). Effects of feeding and maturation system on qualitative characteristics of buffalo meat (Bubalus bubalis). Animals.

[7] Smaldone G., Marrone R., Vollano L., Peruzy M.F., Barone C.M.A., Ambrosio R.L., Anastasio A. (2019). Microbiological, rheological and physical-chemical characteristics of bovine meat subjected to a prolonged ageing period. Ital. J. Food Saf..

[8] Kerth C.R., Miller R.K. (2015). Beef flavor: A review from chemistry to consumer. J. Sci. Food Agric..

[9] Ruan E.D., Aalhus J.L., Juárez M., Sabik H. (2015). Analysis of volatile and flavor compounds in grilled lean beef by stir bar sorptive extraction and thermal desorption—Gas chromatography mass spectrometry. Food Anal. Methods.

[10] Frank D., Oytam Y., Hughes J. (2017). Sensory perceptions and new consumer attitudes to Meat. New Aspects of Meat Quality.

[11] Mottram D.S. (1998). Flavour formation in meat and meat products: A review. Food Chem..

[12] Calkins C.R., Hodgen J.M. (2007). A fresh look at meat flavor. Meat Sci..

[13] Johnson J.A., Sutherland B.D., Mckinnon J.J., Mcallister T.A., Penner G.B. (2019). Use of barley or corn silage when fed with barley, corn, or a blend of barley and corn on growth performance, nutrient utilization, and carcass characteristics of finishing beef cattle. Transl. Anim. Sci..

[14] Lardner H.A., Pearce L., Damiran D. (2017). Evaluation of low heat unit corn hybrids compared to barley for forage yield and quality on the Canadian prairies. Sustain. Agric. Res..

[15] Humer E., Zebeli Q. (2017). Grains in ruminant feeding and potentials to enhance their nutritive and health value by chemical processing. Anim. Feed Sci. Technol..

[16] Nikkhah A. (2012). Barley grain for ruminants: A global treasure or tragedy. J. Anim. Sci. Biotechnol..

[17] Wismer W.V., Okine E.K., Stein A., Seibel M.R., Goonewardene L.A. (2008). Physical and sensory characterization and consumer preference of corn and barley-fed beef. Meat Sci..

[18] Jeremiah L.E., Beauchemin K.A., Jones S.D.M., Gibson L.L., Rode L.M. (1998). The influence of dietary cereal grain source and feed enzymes on the cooking properties and palatability attributes of beef. Can. J. Anim. Sci..

[19] McEwen P.L., Mandell I.B., Brien G., Campbell C.P. (2007). Effects of grain source, silage level, and slaughter weight endpoint on growth performance, carcass characteristics, and meat quality in Angus and Charolais steers. Can. J. Anim. Sci..

[20] Vahmani P., Johnson J.A., Sutherland B.D., Penner G.B., Prieto N., Aalhus J.L., Juárez M., Lopez-Campos O., Dugan M.E.R. (2020). Changes in the fatty acid composition of steer subcutaneous fat, including biohydrogenation products, are minimal when finished on combinations of corn and barley grains and silages. Can. J. Anim. Sci..

[21] Burnett D.D., Legako J.F., Phelps K.J., Gonzalez J.M. (2020). Biology, strategies, and fresh meat consequences of manipulating the fatty acid composition of meat. J. Anim. Sci..

[22] CCAC (2009). Guidelines on: The Care and Use of Farm Animals in Research, Teaching, and Testing.

[23] AMSA, American Meat Science Association (2016). Research Guidelines for Cookery, Sensory Evaluation, and Instrumental Tenderness Measurements of Meat.

[24] Larmond E. (1977). Laboratory Methods for Sensory Evaluation of Foods.

[25] Vahmani P., Rolland D.C., McAllister T.A., Block H.C., Proctor S.D., Guan L.L., Prieto N., López-Campos Ó., Aalhus J.L., Dugan M.E.R. (2017). Effects of feeding steers extruded flaxseed on its own before hay or mixed with hay on animal performance, carcass quality, and meat and hamburger fatty acid composition. Meat Sci..

[26] Lê S., Josse J., Husson F. (2008). FactoMineR: An R package for multivariate analysis. J. Stat. Softw..

[27] ter Braak C.J.F. (1990). Interpreting canonical correlation analysis through biplots of structure correlations and weights. Psychometrika.

[28] Legako J.F., Brooks J.C., O’Quinn T.G., Hagan T.D.J., Polkinghorne R., Farmer L.J., Miller M.F. (2015). Consumer palatability scores and volatile beef flavor compounds of five USDA quality grades and four muscles. Meat Sci..

[29] Song S., Zhang X., Hayat K., Liu P., Jia C., Xia S., Xiao Z., Tian H., Niu Y. (2011). Formation of the beef flavour precursors and their correlation with chemical parameters during the controlled thermal oxidation of tallow. Food Chem..

[30] Larick D.K., Turner B.E. (1990). Headspace volatiles and sensory characteristics of ground beef from forage- and grain-Fed heifers. J. Food Sci..

[31] Tao N.P., Wu R., Zhou P.G., Gu S.Q., Wu W. (2014). Characterization of odor-active compounds in cooked meat of farmed obscure puffer (*Takifugu obscurus*) using gas chromatography-mass spectrometry-olfactometry. J. Food Drug Anal..

[32] Stetzer A.J., Cadwallader K., Singh T.K., Mckeith F.K., Brewer M.S. (2008). Effect of enhancement and ageing on flavor and volatile compounds in various beef muscles. Meat Sci..

[33] Therkildsen M., Spleth P., Lange E.-M., Hedelund P.I. (2017). The flavor of high-quality beef—A review. Acta Agric. Scand. Sect. A Anim. Sci..

[34] Calkins B.C.R., Ph D., Sullivan G. (2006). Ranking of Beef Muscles for Tenderness. https://www.beefcentral.com/wp-content/uploads/2014/05/ranking-of-beef-muscles-for-tenderness.pdf.

[35] Legako J.F., Dinh T.T.N., Miller M.F., Adhikari K., Brooks J.C. (2016). Consumer palatability scores, sensory descriptive attributes, and volatile compounds of grilled beef steaks from three USDA Quality Grades. Meat Sci..

[36] Starowicz M., Zieliński H. (2019). How Maillard reaction influences sensorial properties (color, flavor and texture) of food products?. Food Rev. Int..

[37] Sun W., Zhao M., Cui C., Zhao Q., Yang B. (2010). Effect of Maillard reaction products derived from the hydrolysate of mechanically deboned chicken residue on the antioxidant, textural and sensory properties of Cantonese sausages. Meat Sci..

[38] Migita K., Iiduka T., Tsukamoto K., Sugiura S., Tanaka G., Sakamaki G., Yamamoto Y., Takeshige Y., Miyazawa T., Kojima A. (2017). Retort beef aroma that gives preferable properties to canned beef products and its aroma components. Anim. Sci. J..

[39] Hwang Y.-H., Joo S.-T. (2017). Fatty acid profiles, meat quality, and sensory palatability of grain-fed and grass-fed beef from Han-woo, American, and Australian crossbred cattle. Korean J. Food Sci. Anim..

[40] Wood J.D., Enser M., Fisher A.V., Nute G.R., Sheard P.R., Richardson R.I., Hughes S.I., Whittington F.M. (2008). Fat deposition, fatty acid composition and meat quality: A review. Meat Sci..

[41] Johnson J.A., Sutherland B.D., McKinnon J.J., McAllister T.A., Penner G.B. (2020). Effect of feeding barley or corn silage with dry-rolled barley, corn, or a blend of barley and corn grain on rumen fermentation, total tract digestibility, and nitrogen balance for finishing beef heifers. J. Anim. Sci..

[42] Enjalbert F., Combes S., Zened A., Meynadier A. (2017). Rumen microbiota and dietary fat: A mutual shaping. J. Appl. Microbiol..

[43] Miller R.K., Rockwell L.C., Lunt D.K., Carstens G.E. (1996). Determination of the flavor attributes of cooked beef from cross-bred Angus steers fed corn-or barley-based diets. Meat Sci..

[44] Brassard M.-E., Chouinard Y., Gervais R., Pouliot E., Gariépy C., Cinq-Mars D. (2017). Effects of level of barley and corn in concentrate-fed Boer kids on growth performance, meat quality and muscle fatty acid composition. Can. J. Anim. Sci..

[45] Elmore J.S., Mottram D.S., Enser M., Wood J.D. (1999). Effect of the polyunsaturated fatty acid composition of beef muscle on the profile of aroma volatiles. J. Agric. Food Chem..

[46] Shahidi F., Pegg R.B. (1994). Hexanal as an indicator of meat flavor deterioration. J. Food Lipids.

[47] Nelson M.L., Busboom J.R., Ross C.F., O’Fallon J. (2008). V Effects of supplemental fat on growth performance and quality of beef from steers fed corn finishing diets. J. Anim. Sci..

