# Supplementary material for: Effect of Feeding Barley, Corn, and a Barley/Corn Blend on Beef Composition and End-Product Palatability
Source: Foods. 2021 Apr 29;10(5):977. doi: 10.3390/foods10050977 (PMC8146225; doi:10.3390/foods10050977)
Supplement: Supplementary file 1 [file foods-10-00977-s001.zip › Supplementary Table 1-Volatile No Selected.pdf]

Supplementary Table 1. Mean of standardized base peak of non-selected volatile compounds from barley, corn and blended grain-fed beef samples.

Volatile compounds	Barley	Blended	Corn
**(1-Propyloctyl)benzene**	0	0.00332523	0
**(E,E)-2,4-Heptadienal**	0.05499927	0.04133336	0.03616535
**(E,E)-2,4-Nonadienal**	0.28332775	0.28667742	0.25596278
**(E,Z,Z)-2,4,7-Tridecatrienal**	0.08266665	0.09089641	0.06299861
**(E,Z)-2,6-Nonadienal**	0.01238146	0.01163414	0.01204181
**(E)-2-Nonenal**	0.10572931	0.09820879	0.08462778
**(E)-2-Octen-1-ol**	0.06625213	0.0610077	0.05361802
**(E)-2-Octenal**	0.11104416	0.10242353	0.10114867
**(E)-2-Undecenal**	0.24955702	0.24918295	0.22333874
**(E)-3-Tetradecene**	0.00017808	0.00021921	0.00015502
**(Z)-11-Octadecenoic acid**	0.04269325	0.01924938	0.02756694
**(Z)-9-Hexadecenal**	0.14340133	0.10446274	0.16610462
**1-Butanol**	0.00129414	6.92E-05	0.00086856
**1-Chlorohexane**	3.48E-05	0	9.80E-05
**1-Decanol**	0.07653255	0.07480853	0.07170082
**1-Dodecanol**	0.09016195	0.07951297	0.11441492
**1-Dodecene**	0.01205798	0.01149578	0.0174805
**1-Heptanol**	0.02653987	0.02697966	0.02657724
**1-Hexanol**	0.00498783	0.00420937	0.0068452
**1-Methoxyhexane**	6.16E-05	0	0.00097507
**1-Nonanol**	0.01706481	0.0219253	0.0159335
**1-Nonen-3-ol**	0.00034224	0.0002438	0.00030803
**1-Octanol**	0.12030986	0.15005288	0.10340793
**1-Pentadecanol**	0.11050303	0.09169675	0.09990376
**1-Pentanol**	0.01401083	0.0095234	0.01230314
**1-Tridecanol**	0.05989379	0.06802657	0.07152793
**1-Undecene**	0.00408847	0.00449256	0.00796533
**1,2-Dimethyl naphthalene**	0.00480856	0.00762257	0.00302381
**1,4-Dichlorobenzene**	0.0030849	0.00706748	0.01064631
**1,6,7-Trimethyl naphthalene**	0.00529654	0.0066039	0.00129754
**2-Butylfuran**	0.00029787	1.69E-05	0.00077348
**2-Decanone**	0.05808011	0.08574807	0.10241707
**2-Decenal**	0.30571662	0.3068575	0.25806928
**2-Ethyl-1-hexanol**	0.24878587	0.29594167	0.21564291
**2-Ethyl-3-hydroxyhexyl 2-methylpropanoate**	0.10099326	0.12089483	0.09815403
**2-Heptanone**	0.01773103	0.01700561	0.02372422

**2-Heptenal**	0.04106154	0.03741087	0.04432526
**2-Methyl naphthalene**	0.00217512	0.00292765	0.00158533
**2-Methyl-3-octanone**	0.01234288	0.01661276	0.00026971
**2-Methyldecane**	0.00059393	0	0.00013222
**2-Nonanone**	0.02467708	0.03321998	0.03957208
**2-Octanone**	0.01764542	0.02990792	0.0271451
**2-Pentylfuran**	0.10923132	0.08502066	0.08977243
**2-Pentylpyridine**	0.00056325	0.00333918	0.00301594
**2-Pentylthiazolidine**	0.00182309	0.00483585	0.00206357
**2-Propylfuran**	9.76E-06	4.24E-05	1.67E-05
**2-Tridecanone**	0.01124647	0.01446584	0.00796469
**2,3-Butanediol**	0.0020966	0.00091167	0.0032503
**2,3-Dimethylheptane**	0	0.00030163	0.00014676
**2,3,5-Trimethyl-6-(3-methylbutyl)-pyrazine**	0.01382519	0.01283311	0.00389287
**2,4-Decadienal**	0.07055069	0.05460991	0.05737823
**2,4-Di-tert-butylphenol**	0.06360755	0.06827365	0.04963654
**2,6-Diethylpyrazine**	0.05428039	0.03589811	0.0578025
**3-Ethyl-2,5-dimethylpyrazine**	0.11796493	0.11534369	0.11352501
**3-Ethyltridecane**	0	0	0.00027011
**3-Isopropyl-6-methylcyclohexene**	0.00343067	0.00338338	0.00323647
**3-Methylbutanal**	0.00037408	0.00027464	0.0022048
**3-Methylbutanoic acid**	0.01050637	0.01029879	0.03054749
**3-Methyldecane**	0.02467418	0.02464789	0.02511667
**3-Methylpentadecane**	0.01870563	0.01393278	0.01670807
**3-Nonanone**	0.00107067	0.00171047	0.00305039
**3-Octanone**	0.03496251	0.07828504	0.02919882
**3-Octen-2-one**	0.01596934	0.01564072	0.01527915
**3,5-Octadien-2-one**	0.01354954	0.01053318	0.00766336
**4-Ethylbenzaldehyde**	0.00346906	0.00445898	0.00648113
**4-Methylbenzaldehyde**	0.00769432	0.01143141	0.01130213
**4-Methyloctane**	0.00050061	0	0.00010317
**4-Methylundecane**	0.00840369	0.00658492	0.00354822
**4-Nonenal**	0.00795226	0.00797187	0.00611341
**4-Pentylbenzaldehyde**	0.00038692	0.00036184	0.00032399
**5-Ethyl-1-formylcyclopentene**	0.00281997	0.00220514	0.00234619
**5-Ethyl-2,3-dimethylpyrazine**	0.01887504	0.01377539	0.01614535
**a-Phellandrene**	0.00093466	0.00083367	0.00041687
**a-Thujene**	0.00071281	0.00045411	0.00048278
**alpha-Pinene**	0.00018558	9.48E-05	5.33E-05
**Benzaldehyde**	0.04903391	0.04322561	0.115082

**Benzophenone**	0.10045945	0.10143332	0.08136893
**Butylated hydroxytoluene**	0.03916871	0.03909776	0.03862474
**Butylbenzene**	0.00103687	0.00105527	0.00064697
**Cumene**	0.00016619	6.81E-05	0.01045251
**Cuminaldehyde**	0.04257936	0.03255089	0.05625314
**Decanal**	0.15972425	0.20421371	0.19099754
**Decane**	0.08287893	0.06827568	0.06984981
**delta-Dodecalactone**	0.13047779	0.14145568	0.17644468
**Diethyl phthalate**	0.00119453	0.03295124	0.01826625
**Dodecanal**	0.04529935	0.06101603	0.06012344
**Dodecane**	0.11172172	0.10632746	0.13376063
**Ethylbenzene**	0.00073006	0.00577932	0.00338862
**gamma-Dodecalactone**	0.00198574	0.01017237	0.01459433
**Geranyl acetone**	0.00524871	0.00345733	0.00299319
**Heptanal**	0.07394428	0.06547291	0.08355403
**Hexadecanol**	0.08064339	0.05263048	0.05274285
**Hexanoic acid**	0.0533213	0.04415529	0.04103461
**Lavender lactone**	0.00037125	0.00011406	0.00075154
**Limonene**	0.01505815	0.01563024	0.01573955
**Linoleic acid**	0.30903734	0.33055693	0.3196548
**m-Cymene**	0.00049359	0.00073274	0.0006557
**m-Di-tert-butylbenzene**	0.13545305	0.22965784	0.15466738
**Menthol**	0.01031245	0.00923616	0.00764156
**Methyl 14-methylpentadecanoate**	0	0	0.01540491
**Methyl 2-methylbutanoate**	0	0.00014216	0.00012372
**Methyl butanoate**	4.47E-05	0.00033557	0.00024663
**Methyl heptadecanoate**	0.03502186	0.00487121	0.01967373
**Methyl heptanoate**	0	0.00063092	0.00201756
**Methyl heptenone**	0.00522021	0.00627568	0.00766315
**Methyl hexadecanoate**	0.01018253	0.02315767	0.07201593
**Methyl hexanoate**	0.00029073	0.00039229	0.00025951
**Methyl methacrylate**	0.00158871	0	0
**Methyl nonanoate**	0	0.00046903	0.00036017
**n-Octyl-cyclopropane**	0.00593461	0.00998917	0.01253052
**N,N-dibutylformamide**	0.01391581	0.04061697	0.0114125
**Naphthalene**	0.0014595	0.00382006	0.00612689
**Nerylacetone**	0.14072093	0.14940974	0.14007209
**Nonanal**	1	1	1
**Nonane**	0.0246675	0.04101522	0.02512359
**Nonanoic acid**	0.06134177	0.05133886	0.05411756

**o-Xylene**	0.12194869	0.22104332	0.07254382
**Octanal**	0.1778603	0.16451656	0.17346796
**Octane**	0.02903676	0.0337197	0.04397178
**Octylbenzene**	0.00013631	0	0
**p-Cymene**	0.00070204	0.00066704	0.03740108
**p-Cymenene**	0.00011324	0.00022223	0.00107483
**p-Menth-1-ene**	8.43E-05	5.94E-05	0.00019037
**p-Xylene**	0	6.08E-05	0
**Pentadecanal**	0	0.00827225	0.0041366
**Pentadecane**	0.34489306	0.31620468	0.29745938
**Pentanal**	0.01111604	0.00632123	0.01562523
**Piperitone**	0.00871853	7.90E-05	0.00330094
**Styrene**	0.00620501	0.00273912	0.06894263
**Sylvestrene**	0.00469711	0.00658357	0.0044721
**Tetradecanal**	0.39282653	0.30925241	0.33889347
**Tetradecane**	0.40207969	0.44571282	0.39424631
**Tetradecanoic acid**	0	0.00280659	0.00015315
**Thiazole**	0	0.0002258	6.35E-05
**Toluene**	0.00328304	0.00254391	0.00526583
**Tributyl phosphate**	0.35034535	0.36013174	0.33103306
**Tridecanal**	0.24511573	0.20445514	0.19509785
**Tridecane**	0.08486665	0.09479349	0.09177113
**Trimethylpyrazine**	0.08439273	0.07161438	0.07376284
**Undecanal**	0.06974575	0.08943401	0.08598671
