# Supplementary material for: Effect of Feeding Barley, Corn, and a Barley/Corn Blend on Beef Composition and End-Product Palatability
Source: Foods. 2021 Apr 29;10(5):977. doi: 10.3390/foods10050977 (PMC8146225; doi:10.3390/foods10050977)
Supplement: Supplementary file 1 [file foods-10-00977-s001.zip › Supplementary table S2-Sensory-Flavour.pdf]

Supplementary table 2. Mean and standard deviation of sensory descriptive and flavour profile attributes from barley, corn and blended grain-fed beef samples.

	Barley	Blended	Corn
Sensory descriptive attributes			
Initial Tenderness	6.15±1.31	5.94±1.37	6.35±1.03
Initial Juiciness	5.06±1.09	4.86±1.21	4.55±0.99
Beef Flavour Intensity	5.29±0.64	5.89±0.62	5.46±0.60
Off-Flavour Intensity	7.20±1.23	7.65±1.49	7.75±1.24
Connective Tissue	8.36±1.23	8.01±1.03	8.33±0.51
Overall Tenderness	6.58±1.11	6.37±1.23	6.62±0.89
Sustainable Juiciness	5.10±0.72	5.08±0.70	4.83±0.77
Flavour profile			
Aroma attributes			
Beef identity	4.76±0.80	4.68±0.88	4.80±0.82
Barnyard	2.50±0.82	2.12±0.62	2.63±0.71
Bloody-serumy	1.88±1.09	2.05±0.48	2.02±0.79
Brown-roasted	3.83±0.73	3.82±0.60	3.60±0.92
Burnt	2.40±0.69	2.82±0.71	2.07±0.69
Buttery	2.94±0.42	2.62±0.64	2.69±0.50
Corn	2.35±1.14	1.89±0.61	2.26±1.16
Cruciferous	2.52±0.61	2.76±1.03	2.66±1.13
Fat-like	2.49±0.51	2.41±0.50	2.28±0.66
Grainy	2.04±0.85	2.21±0.75	1.86±0.65
Green-hay	2.34±0.98	2.72±0.85	2.72±0.95
Liver-like	2.06±1.32	2.17±0.88	2.73±1.00
Metallic	1.66±0.32	2.15±1.82	2.54±1.20
Sour-dairy	2.78±1.16	3.40±1.19	2.50±0.47
Stale-cardboard	2.63±0.81	3.00±2.40	-
Other	3.72±0.41	4.56±2.12	4.32±1.47
Unidentified	4.25±1.46	3.86±0.83	3.82±1.84
Taste attributes			
Salty	2.45±0.51	2.36±0.47	2.45±0.49
Sour	3.42±0.87	3.55±0.87	3.42±0.87
Sweet	3.00±1.76	3.02±1.74	3.00±1.33
Bitter	2.48±0.85	2.46±0.70	2.48±0.87
Umami	2.84±0.64	2.77±0.60	2.84±0.58
Flavour attributes			
Beef identity	5.07±0.70	5.33±0.60	5.45±0.64
Barnyard	2.74±1.29	2.39±0.37	2.48±0.30

Bloody-serumy	3.48±1.47	3.05±1.45	2.88±1.26
Brown-roasted	3.22±0.62	3.23±0.74	3.40±0.68
Burnt	3.75±0.91	3.60±2.22	2.20±0.14
Buttery	2.43±0.61	2.38±0.53	2.48±0.53
Corn	2.07±0.92	2.19±0.56	1.84±0.63
Cruciferous	2.38±0.74	2.83±0.87	2.42±1.10
Fat-like	2.55±0.61	2.51±0.43	2.36±0.82
Grainy	2.08±0.89	2.03±0.57	2.22±0.82
Green-hay	5.06±1.41	4.56±1.32	4.50±2.01
Liver-like	2.60±1.29	2.92±1.29	2.71±1.38
Metallic	2.42±0.86	1.90±1.02	2.24±1.09
Oily	1.25±0.07	1.52±0.53	2.30±0.45
Rancid	-	2.13±0.65	1.6
Sour-dairy	4.10±1.17	3.96±1.17	3.71±1.21
Stale-cardboard	3.15±1.46	2.93±1.45	3.21±1.50
Other	4.50±1.55	6.05±2.33	3.1
Unidentified	4.69±0.89	3.95±0.61	4.38±1.51
